# Complete Response of Synchronous Liver Metastasis in a Pancreatic Ductal Adenocarcinoma, When Surgery Could Represent a Therapeutic Option

**DOI:** 10.1155/2020/8679751

**Published:** 2020-10-09

**Authors:** Antonella Argentiero, Angela Calabrese, Angela Monica Sciacovelli, Sabina Delcuratolo, Antonio Giovanni Solimando, Oronzo Brunetti

**Affiliations:** ^1^Medical Oncology Unit, IRCCS Istituto Tumori “Giovanni Paolo II”, Bari, Italy; ^2^Radiology Unit, IRCCS Istituto Tumori “Giovanni Paolo II”, Bari, Italy; ^3^Department of Biomedical Sciences and Human Oncology, Section of Internal Medicine “G. Baccelli”, University of Bari Medical School, Bari, Italy

## Abstract

Metastatic pancreatic ductal adenocarcinoma (PDAC) is characterized by poor prognosis and short survival. Today, the use of new polytherapeutic regimens increases clinical outcome of these patients opening new clinical scenario. A crucial issue related to the actual improvement achieved with these new regimens is represented by the occasional possibility to observe a radiological complete response of metastatic lesions in patients with synchronous primary tumor. What could be the best therapeutic management of these patients? Could surgery represent an indication? Herein, we reported a case of a patient with PDAC of the head with multiple liver metastases, who underwent first-line chemotherapy with mFOLFIRINOX. After 10 cycles, he achieved a complete radiological response of liver metastases and a partial response of pancreatic lesion. A duodenocephalopancreasectomy was performed. Due to liver a lung metastases after 8 months from surgery, a second-line therapy was started with a disease-free survival and overall survival of 8 months and 45 months, respectively. Improvement in the molecular characterization of PDAC could help in the selection of patients suitable for multimodal treatments. This trial is registered with NCT02892305 and NCT00855634.

## 1. Introduction

Pancreatic ductal adenocarcinoma (PDAC) is one of the most important issues in the context of cancer being the fourth leading cause of death in USA and Japan and the sixth in Europe [1, 2] with a 5-year survival no greater than 6% [2] and an estimated increase in incidence that will bring it to the second leading cause of cancer death in 2030 [3]. At first diagnosis, only from 10% to 20% of PDAC patients present a primarily resectable disease. Approximately, 60% of patients are affected by metastatic disease [4].

Today, according to international guidelines, distant metastases (including nonregional lymph nodes) and vascular infiltration are absolute contraindication to surgery [5]. Surgical resection of PDAC with synchronous distant metastases is not indicated as the average survival time which appears equivalent to that of chemotherapy alone [6].

In the past, in the absence of active primary chemotherapy regimens, many surgeons attempted to resect liver or lymph node metastasis in a single operation or in two different times after resection of the primary with detrimental results in terms of survival and quality of life [7].

Today, the use of polychemotherapy regimens increases the chemosensitivity and the rate of response to the disease. In a phase III randomized study, the combination chemotherapy FOLFIRINOX gained a significant advantage in terms of progression-free survival (PFS) (6.4 months versus 3.3 months; *p* < 0.0001) and overall survival (OS) (11.1 months versus 6.8 months, 1-y OS versus 48.4%, 20.6%; *p* < 0.0001) compared to gemcitabine in patients with metastatic disease and age ≤70 years [8]. Furthermore, another randomized phase III study of 861 patients with mPDAC previously untreated have shown that the combination gemcitabine-nabpaclitaxel can improve PFS (HR 0.69; *p* < 0.0001) and OS (HR 0.72; *p* < 0.0001) compared to gemcitabine alone [9].

A crucial issue related to the actual improvement achieved with these new regimens is represented by the occasional possibility to observe a radiological complete response of metastatic lesions in patients with synchronous primary tumor [10]. What could be the best therapeutic management of these patients? Could surgery represent an indication? Herein, we discuss the role of surgery in a long-term metastatic PDAC survivor who presented a complete response of synchronous liver metastases after modified FOLFIRINOX regimen.

## 2. Case Report

**Figure 1:**
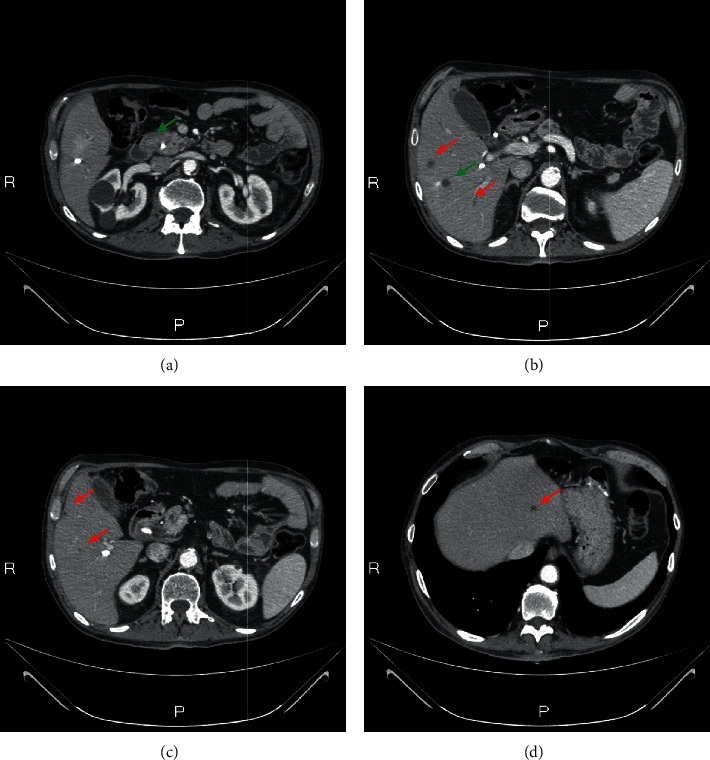
Staging: radiological evaluation of primary pancreatic lesion (a) and liver metastasis (b–d). The green arrows indicate the biopsied lesions and the red arrows indicate the liver lesions.

**Figure 2:**
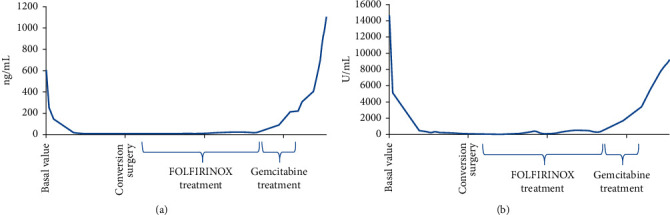
Trend of neoplastic markers: CEA (a) and Ca 19.9 (b).

**Figure 3:**
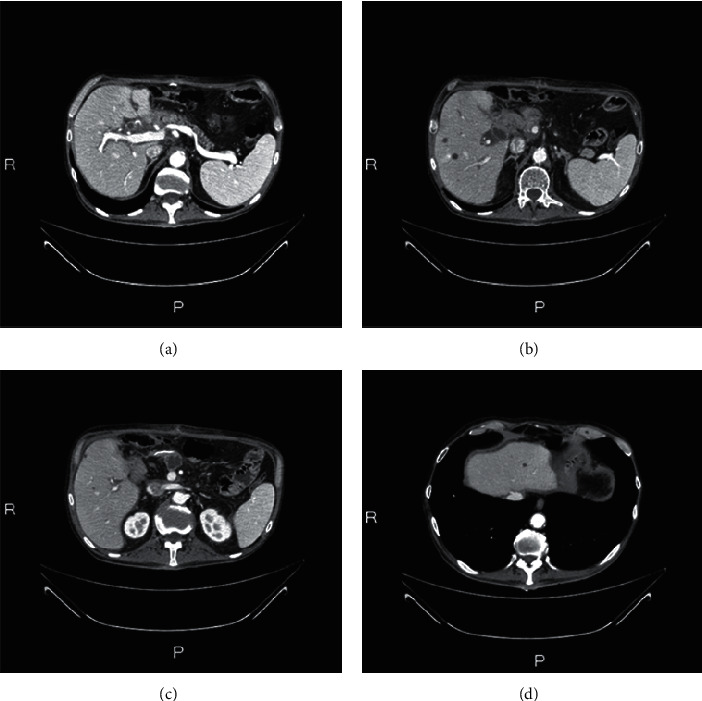
Radiological evaluation before surgery: primary pancreatic lesion (a) and liver metastasis (b–d).

A Caucasian 64-year-old man suffering from hypertension and diabetes presented with a history of abdominal pain in the last two months. A computed tomography (CT) scan of the abdomen revealed a lesion of 4 cm in diameter localized in the head of pancreas with the presence of venous involvement of the superior mesenteric vein (Figure 1(a)) without a clear cleavage plane from the descending part of the duodenum and an initial dilatation of the intrahepatic bile duct. Six secondary liver lesions were concomitant (Figures 1(b)–1(d)). In particular, 2 of these lesions ranged from 1 to 2 cm and the other 4 were millimetric ones; so far, we consider it as an oligometastatic cancer. Serum levels of CEA and Ca 19.9 were 721 ng/mL and 11.200 U/mL, respectively (Figures 2(a) and 2(b)). A fine-needle biopsy of both pancreatic and the V segment lesion of the liver reported the diagnosis of malignant cells compatible with moderately differentiated PDAC. First-line chemotherapy according to the modified FOLFIRINOX regimen (oxaliplatin 85 mg/m^2^, irinotecan 180 mg/m^2^, folinic acid 400 mg/m^2^, continuous 44 hours infusion of 5FU 2400 mg/m^2^, every 2 weeks) was started. After 4 cycles, a CT scan of the abdomen documented a partial response of all target lesions according to RECIST 1.1 criteria [11]. Nevertheless, due to the appearance of a subocclusive clinical scenario associated with an acute bacterial pneumonia and a rapid decay of performance status, the patient underwent an esophageal-gastric-duodenum endoscopy showing duodenal stenosis associated with severe gastric displacement. Therefore, a palliative gastro-entero-anastomosis was performed associated with a targeted antibiotic therapy for pneumonia. In the following 3 months, only best-supportive care was initiated in order to improve patient's health status. At that time, a new CT scan documented the increase of both pancreatic and liver lesions, serum tumor markers, and obstructive jaundice with high total bilirubin levels (15.7 mg/dL). As a consequence, a biliary drainage was implanted with a rapid restoration of normal bilirubin values. In the next month, the FOLFIRINOX regimen was resumed and 6 cycles were administered without significant toxicities. A progressive decline of CEA and Ca 19.9 was achieved with values of 110 ng/mL and 152 U/mL, respectively. A new CT scan showed dimensional stability of both hepatic and pancreatic lesions associated with a prevalence of necrotic areas (Figures 3(a)–3(d)). Simultaneously, a PET was negative. After one month, an exploratory laparotomy was performed. Intraoperative extemporaneous histological examination of macrobiopsy of two liver lesions at the V segment was negative for malignancy. Thus, a duodenocephalopancreasectomy was performed. Microscopic examination reported the diagnosis of PDAC with negative surgical margins and 7 out of 25 metastatic pancreatic lymph nodes (ypT2N1). After 2 months, a CT scan of the chest and abdomen showed no secondary lesions. ASCO guidelines recommend a total of 6 mounts CT between neoadjuvant and adjuvant chemotherapy. So far, no systemic chemotherapy was administered in the following 6 months. Next, two consecutive CT scans identified no metastases, showing only three stable subcentimetric liver nodules. During this period, a slow progressive increase of tumor markers was documented.

**Figure 4:**
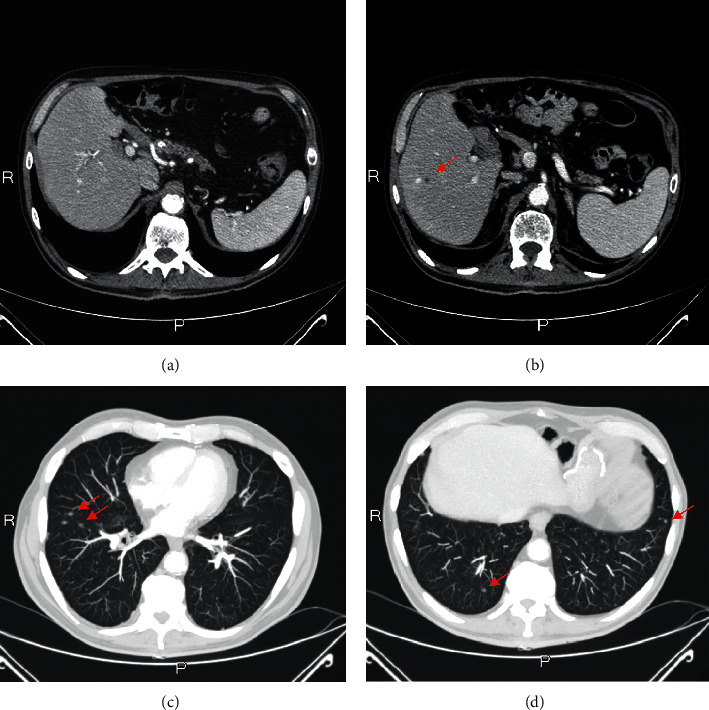
Disease relapse: radiological evaluation of primary pancreatic lesion (a), liver (b), and lung metastasis (c-d). The red arrows indicate the metastatic lesions.

At the third radiological evaluation after surgery, the liver and lung relapse was observed (Figures 4(a)–4(d)). The patient underwent administration of other 20 cycles of mFOLFIRINOX with a 1-year progression-free survival. The most frequently observed mFOLFIRINOX-related grade 1-2 adverse events were diarrhea, stomatitis, and anemia. Occasionally, grade 3 neutropenia, anemia, diarrhea, and fatigue required dosage adaptions. After that, a second line with gemcitabine was administered for 6 months due to the increase of liver and lung metastases. Overall, the patient achieved an OS of 45 months.

## 3. Discussion

Liver metastases strongly impact PDAC outcome and embody an unmet clinical need target, representing one of the main morbidity and mortality factors in these patients. However, sometimes liver metastases are sensitive to chemotherapy treatment due to greater drug delivery than pancreatic tissue [12]. Unfortunately, the clinical setting of a complete radiological response to liver metastases from PDAC is not supported by high-quality literature data. So far, the specialist has no strong evidences for clinical judgment-based therapeutic decision [13].

Indeed, only scanty evidences derived from case reports and retrospective analyses have been published to date [14]. Limitations of these studies are the retrospective methodology employed, the population heterogeneity, the difference between the surgery volume of the referral centers involved, and the lack of homogeneity of the primary cytoreductive therapy utilized. Moreover, the concordance between experts in the field is also insufficient. Despite the available evidences do not support upfront synchronous resection of PDAC liver metastases, conversion surgery after optimal response to chemotherapy justifies a reasonable optimism for such integrated therapeutic window [14]. It is rational to include our case clinical course within the PDAC phenotype identified by Frigerio et al., in which the complete response obtained on liver metastases to a first-line cytoreduction might predict a favorable clinical outcome with a median overall survival (OS) of 56 months for 24 out of 535 subjects (4.5%) bridged to surgery. The regimen employed was either FOLFIRINOX (66%) or gemcitabine-based therapy (34%) [15], leading to 88% of R0 resection and to 17% of patients gaining a complete pathological response. The mortality rate was 0%. Furthermore, also primary tumor excision along with synchronous metastatic surgical resection for 23 patients out of 1147 (2%) in optimal response after either FOLFIRINOX (61%) or gemcitabine-based therapy (39%) showed a median OS of 34.1 months [16]. In light of the aforementioned data, further reports confirmed analogous clinical behavior [17].

Analogous reports are derived from Crippa et al. [18], who published the results of a retrospective bi-institutional study on the role of surgery in patients with liver metastatic PDAC with good performance status who underwent primary chemotherapy with subsequent radiological response and biochemistry. The study included 127 patients who underwent various chemotherapy schemes: gemcitabine-based (44%); FOLFIRINOX (8%); cisplatin, gemcitabine, capecitabine, and epirubicin (PEXG)/capecitabine and docetaxel (PDXG)/epirubicin and fluorouracil (PEFG) (48%). 56 patients (44%) had a complete (7%) or partial (37%) metastasis radiological response. Surgical treatment was considered in patients with complete or partial radiological response and with normalization of CA 19.9 or reduction of CA 19.9 >90% compared to the initial value. 11 patients (8.5%) underwent surgical resection. Median OS was 11 months in the entire cohort and 15 months for patients with complete/partial response. In this subgroup, OS was significantly longer in patients undergoing surgical resection (median OS: 46 months versus 11 months; *p* < 0.0001). Some authors identified the following as independent survival factors: multichemotherapy (HR: 0.512), surgical resection (HR: 0.360), >5 liver metastases at diagnosis (HR: 3.515), and reduction of CA 19.9 <50% compared to diagnosis (HR: 2.708). A retrospective analysis and a low number of patients undergoing surgical resection affect the study methodology. Nonetheless, the data obtained inspired further well-designed statistically powered clinical trial (i.e., ClinicalTrials.gov identifier: NCT02892305 and NCT00855634). Indeed, Crippa et al. highlighted the fundamental role of patient selection in driving the therapeutic strategy, taking into account risk factors, cytoreductive regimen employed, and prognostic determinants such as the radiological and biochemical responses [18].

Conversely, some authors showed that synchronous pancreatic and liver metastases resection upfront did not result in improved survival compared to palliative treatment (mOS range of 6 months) and does not appear to be justified [6, 19].

Other evidences reported a small increase in survival for resection of synchronous PDAC liver metastases with acceptable safety in highly selected patients [20, 21]. Hackert et al. published the results of a single-center retrospective study in which postoperative complications and survival were evaluated in 62 patients with PDAC with synchronous liver metastases undergoing pancreatic and hepatic primary surgical resection. Patients suffered from limited liver disease (oligometastatic pancreatic cancer) and in 57 patients an atypical liver resection of one or two metastases was performed. About 10% of patients developed a clinically significant pancreatic fistula, 6.4% postoperative bleeding; 3.2% of patients underwent second-surgery and 30-day mortality was 1.6%. Median OS was 12.3 months and 5-year survival was 8.1%. Limitations of this study consisted of retrospective analysis and the lack of complete data regarding the adjuvant treatment employed [22].

Therefore, according to current evidences, it is reasonable to suggest that in patients with liver oligometastatic PDAC cancer, surgery upfront indication would necessitate prospective controlled clinical trials to support clinical decisions.

Conversely, surgical treatment can be considered in highly selected metastatic PDAC cases with stringent response to primary chemotherapy in clinical trials at reference centers. However, to date, there are no selection criteria for primitive or liver metastasis resection of mPDAC.

Given the presented elements, it would be of paramount importance to identify two orders of criteria aimed to properly tailor the combination approach to mPDAC: biologic predictors might foster a personalized therapeutic plan and imaging criteria, able to resolve the response criteria dilemma and to hold the promise to dissect the potential cure rate of a given patient subgroup. In some carefully selected cases after primary chemotherapy, the objective response assessment by imaging and tumor markers can orientate the surgery choice.

Our case report highlights an extraordinary and apparently unpredictable disease course, arising unsolved clinical and preclinical questions. Given that the complete response of hepatic metastasis in PDAC constitutes a rare event, an extensive biologic investigation can help to deeper characterize the underlying unsolved biologic phenotype. The genomic landscape appears to be one of the major challenging factors driving tumor heterogeneity [23, 24]. Both distant metastases [25, 26], nodal involvement [27, 28], and drug resistance [29–31] have been correlated with peculiar molecular signatures in PDAC. Cancer omics and biological signatures are able to stratify tumors depending on the cancer cell phenotype and the tumor niche, able to educate a neoplastic-friendly microenvironment for both solid and hematological cancer [32–36]. Resolving the spatial and clonal cancer heterogeneity might provide fundamental clues, able to deeper characterize translational target and oncogenic drivers, providing novel theragnostic targets.

The paradigm learned from colorectal carcinoma represents a pragmatic integration between biological prognostic factors and progressive resolution of comprehensive surgical-medical approach of metastatic colon carcinoma [37, 38]. The lesson from these evidences drove expanded indication for surgery in metastatic neuroendocrine [39] and renal cell carcinoma [40]. On the contrary, current guidelines do not support surgical approach for PDAC in metastatic setting [5].

There are other reports for conversion therapy for PDAC. There are other reports on mFOLFIRINOX long-term survival in PDAC. To our knowledge, this is the first report of coexistence of prolonged chemotherapeutic exposure along with clinical favorable outcome for a metastatic PDAC patient. In particular, the peculiarity of this report was given to achieving an OS of more than 40 months with a combined strategy of a conversion treatment of a metastatic PDAC patient with mFOLFIRINOX and a long-term administration of this treatment. He complained several related adverse events; nonetheless, we were able to administer more than 30 treatment cycles. Safety profile was acceptable in terms of supportive treatment. Even if this PDAC seemed to be a platinum-sensitive tumor, there was no family history of cancer among relatives of this patient. In any case, it was a BRCA wild type tumor. This multidimensional management displays paramount relevance, taking into account the frequent correlation between the length of treatment and the appearance of AE, which sometimes could require hospitalization [41, 42]. In frame of this thinking, the need of optimal patient selection would prevent unnecessary and unethical treatment, bridging the gap of stratified approach dedicated to subjects harboring clinical and biological signatures that predict more favorable outcome when approached with combined strategies [43, 44].

## Data Availability

The data used to support the findings of this study are included within the article and are available from the corresponding author upon request.
